# Kinetic studies on recombinant UDP-glucose: sterol 3-*O*-β-glycosyltransferase from *Micromonospora rhodorangea* and its bioconversion potential

**DOI:** 10.1186/s13568-016-0224-x

**Published:** 2016-08-02

**Authors:** Nguyen Huu Hoang, Nguyen Lan Huong, Byul Kim, Je Won Park

**Affiliations:** 1 Department of Biotechnology Convergent Pharmaceutical Engineering, SunMoon University, Asan, Chungnam 31460 Republic of Korea; 2 School of Biosystem and Biomedical Science, Korea University, Seoul, 02841 Republic of Korea

**Keywords:** UDP-glucose sterol glycosyltransferase, Kinetics, Fucosterol-3-*O*-β-d-glucoside, Gramisterol-3-*O*-β-d-glucoside, Catalytic promiscuity

## Abstract

Kinetics of a recombinant uridine diphosphate-glucose: sterol glycosyltransferase from *Micromonospora rhodorangea* ATCC 27932 (MrSGT) were studied using a number of sterols (including phytosterols) as glycosyl acceptors. The lowest *K*_m_ value and the highest catalytical efficiency (*k*_cat_/*K*_m_) were found when β-sitosterol was the glycosyl acceptor in the enzymatic reaction. In contrast to the enzyme’s flexibility toward the glycosyl acceptor substrate, this recombinant enzyme was highly specific to uridine diphosphate (UDP)-glucose as the donor substrate. Besides, the UDP-glucose-dependent MrSGT was able to attach one glucose moiety specifically onto the C-3 hydroxyl group of other phytosterols such as fucosterol and gramisterol, yielding stereo-specific fucosterol-3-*O*-β-d-glucoside and gramisterol-3-*O*-β-d-glucoside, respectively. Based on kinetic data obtained from the enzyme’s reactions using five different sterol substrates, the significance of the alkene (or ethylidene) side chains on the C-24 position in the sterol scaffolds was described and the possible relationship between the substrate structure and enzyme activity was discussed. This is the first report on the enzymatic bioconversion of the above two phytosteryl 3-*O*-β-glucosides, as well as on the discovery of a stereospecific bacterial SGT which can attach a glucose moiety in β-conformation at the C-3 hydroxyl group of diverse sterols, thus highlighting the catalytic potential of this promiscuous glycosyltransferase to expand the structural diversity of steryl glucosides.

## Introduction

Uridine diphosphate (UDP)-glucose (Glc) sterol glycosyltransferases (SGTs) belong to family 1 of the 97 families of glycosyltransferases (GTs), which catalyze the transfer of the sugar moiety from the nucleotide activated glycosyl donors onto the nucleophile acceptors (http://www.cazy.org). SGTs are known for the glycosylation of specific acceptors such as sterols (including steroids and steroidal alkaloids), yielding their respective glycosides (Paquette et al. 2003; Chaturvedi et al. 2011; Stucky et al. 2015). They are ubiquitously present in diverse eukaryotic organisms (mainly in plants), and the presence of their catalytic products—phytosterol glycosides (PSGs)—in these organisms might be responsible for physiological functions such as the adaptive responses against biotic or abiotic stresses (Madina et al. 2007; Chaturvedi et al. 2011; Shin et al. 2012; Saema et al. 2016).

A free C-3 hydroxyl position in the phytosterols has been the most preferable and acceptable site for the SGT’s catalytic biosynthesis of PSGs (Tiwari et al. 2014), followed by other hydroxyl groups on the side chain of sterol scaffolds. Some SGTs specifically transfer the sugar moiety onto hydroxyl groups other than the one on C-3 (Madina et al. 2007; Malik et al. 2013). In addition, the stereo-chemical conformation of *O*-glycosidic bond present in the PSGs was mainly in β-conformation (Chaturvedi et al. 2011; Tiwari et al. 2014).

There have been several studies reviewing the catalytic and biochemical properties of plant SGTs, and the structural features and the biological functions of PSGs biosynthesized by these enzymes (Chaturvedi et al. 2011). However, studies on bacterial SGTs have been relatively fewer (Smith 1971; Wunder et al. 2006; Lebrun et al. 2006; Thuan et al. 2013). SGT from *Helicobacter* (a causative agent of peptic or stomach ulcer) glycosylates host cholesterol, thus causing this glycoside to be presented on the outer cell membrane, which in turn confers resistance against the host immune response (Wunder et al. 2006). In 2013, one putative SGT-encoding gene was isolated from a marine actinomycete, *Salinispora tropica* CNB-440; the complete genome sequence of this organism has been published (GenBank Accession Number NC_009380.1). After the heterologous expression of the cloned gene in *Escherichia coli*, the in vitro catalytic function of the gene products has been verified as that of SGT (Thuan et al. 2013).

Recently, we isolated and sequenced an open reading frame expected to encode SGT, from the fosmid libraries of *Micromonospora rhodorangea* ATCC 27932 (deposited as GenBank Accession Number KT983252), using specific degenerate PCR primers (Hoang et al. 2016). The recombinant gene product, MrSGT, expressed in *E. coli* BL21(DE3) as a His-tagged protein, was shown to be capable of transferring a glucosyl moiety from UDP-Glc to the free C-3 hydroxyl position of phytosterols (including β-sitosterol and campesterol) and cholesterol. More detailed kinetic studies of this MrSGT, using a number of sterol substrates, are essential for further investigation of its applicability as a potential tailoring biocatalyst.

In this study, the substrate flexibility of the recombinant MrSGT and the substrate structure-enzyme activity relationship are examined through the comparative kinetic analyses of the purified MrSGT enzyme action on different sterol substrates. In addition, two new PSG derivatives of fucosterol and gramisterol, whose structures have not been described before, are biosynthesized in vitro using MrSGT.

## Materials and methods

### Kinetic analyses of MrSGT

**Fig. 1:**
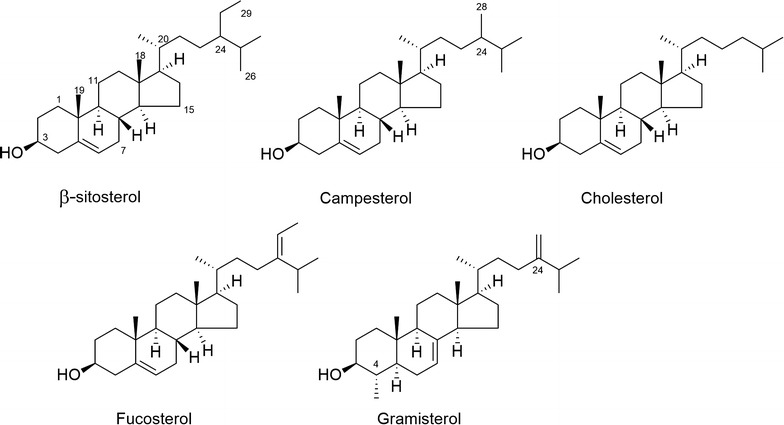
Chemical structures of sterols used as acceptors for in vitro MrSGT enzyme reactions

The purified His-tagged recombinant MrSGT was prepared as previously described in our recent publication (Hoang et al. 2016). The authentic sterols such as β-sitosterol, campesterol and cholesterol (Fig. 1) were purchased from Sigma-Aldrich (St. Louis, MO, USA). MrSGT (2 μM) was incubated with each of the above three sterols (glycosyl acceptors) and UDP-Glc (Sigma-Aldrich) as a glycosyl donor, in 100 μL reaction buffer (50 mM Tris–HCl, pH 7.4; 5 mM MgCl_2_) at 30 °C for 15 min. For the determination of the kinetic parameters for each sterol, the concentration of UDP-Glc was fixed at 0.8 mM, while the concentration of the sterol was varied from 0.05 to 0.6 mM. To measure the kinetic properties with respect to UDP-Glc, the concentration of the sterol was kept constant at 0.6 mM, while the concentration of UDP-Glc was varied from 0.1 to 0.8 mM. All reactions were quenched by the addition of 100 μL of ethyl acetate. After the evaporation of the solvent layer to dryness, aliquots of extracts, reconstituted in methanol, were subjected to HPLC–MS/MS analyses as described below. Each experiment was performed in triplicate, and the reaction mixture containing boiled MrSGT served as control.

### HPLC–MS/MS analyses

Qualitative analyses of the sterols and sterol glucosides (including β-sitosterol-3-*O*-β-d-glucoside, campesterol-3-*O*-β-d-glucoside and cholesterol-3-*O*-β-d-glucoside) obtained from the in vitro MrSGT reactions were conducted by HPLC-tandem mass spectrometry (MS/MS; ThermoFinnigan, San Jose, CA, USA) as described before (Hoang et al. 2016). In brief, isocratic elution (methanol:acetonitrile:water:formic acid = 45:40:14.8:0.2 [v/v/v/v]) was performed on an Acquity CSH C_18_ reversed-phase column (Waters, Milford, MA, USA; 2.1 × 50 mm, 1.7 μm) at a flow rate of 120 μL/min. The column effluent was introduced into the MS/MS (without splitting), which was operated in the positive ion mode. Acquisition was performed using MS/MS operated in the selective reaction monitoring (SRM) mode by choosing six different sets of mass transitions specific to both sterols and the corresponding sterol glucosides to detect the transition of the protonated precursor ion to the dominant product ion (415.5 [M+H]^+^ > 397.5 [M-H_2_O+H]^+^ as a dehydrated product ion for β-sitosterol; 401.5 > 383.5 for campesterol; 387.5 > 369.5 for cholesterol; 577.5 > 399.5 [M-Glc+H]^+^ as an aglycone product ion for β-sitosterol-3-*O*-β-dglucoside; 563.5 > 385.5 for campesterol-3-*O*-β-d-glucoside; 549.5 > 371.5 for cholesterol-3-*O*-β-d-glucoside).

### Glucosylation of fucosterol and gramisterol using MrSGT

The glucosylation of two PSs, whose glucosides have not been exactly described, was examined using purified MrSGT. Authentic fucosterol was obtained from Sigma-Aldrich and authentic gramisterol from Leancare Ltd. (Flintshire, UK) (Fig. 1). Reactions to determine the glycosyltransferase activity of MrSGT on fucosterol and gramisterol, were performed as follows; MrSGT dissolved in the above-mentioned reaction buffer was incubated with 0.6 mM each of fucosterol and gramisterol, respectively, along with 0.8 mM of UDP-Glc at 30 °C for 15 min, and then extracted by organic solvent partition. The formation of the corresponding glucosides was analyzed by HPLC–MS/MS as stated before. Each experiment was performed in duplicate, and the reaction mixture with boiled MrSGT served as control.

### Isolation and structural elucidation of two new PSG derivatives biosynthesized

To produce sufficient quantities of the two PSG derivatives for the purpose of elucidating their chemical structure, multiple scale-up reactions were carried out as follows; the molar concentrations of PSs, UDP-Glc and MrSGT enzyme were maintained in the proportions described above, while scaling up the total volume of the reaction mixture to 1 mL. At the end of the reaction, reaction mixtures from more than five batches were pooled and extracted by ethyl acetate partitioning. The solvent layer obtained was evaporated to dryness using a centrifugal evaporator (EYELA, Tokyo, Japan) set at 40 °C, and reconstituted in 5 mL of the mobile phase used for the HPLC–MS/MS analyses. The extracts were immediately loaded onto a reverse-phase C_8_ cartridge of the CombiFlash Rf medium-pressure liquid chromatography (MPLC) system (Teledyne ISCO, Lincoln, NE, USA), and the flow rate was set at 8 mL/min. The eluents passing through a UV detector were all automatically fractionated over a 50-min running time. The fractions confirmed to contain the PSG derivative with a good purity (>97 %) by the tracing HPLC–MS/MS analyses, were pooled and freeze-dried. Their structures were further confirmed by Varian INOVA 500 nuclear magnetic resonance (NMR, Varian Inc., Palo Alto, CA, USA) spectroscopic analysis together with high resolution (HR) LCT-premier XE MS (Waters, Milford, MA, USA) analysis.

## Results

### Physiological optimum of MrSGT

Prior to examining the kinetic parameters of recombinant MrSGT, the optimum pH and temperature for the glycosyl-transferring reaction of the enzyme were investigated using β-sitosterol as the sterol acceptor, which has been determined in a previous publication (Hoang et al. 2016), to exhibit the highest conversion yield among three different sterols. The enzyme might be stable (>90 % relative activity) within the pH range of 6–8, but its catalytic activity was substantially lost in further alkaline conditions (data not shown). When the temperature was varied between 20 and 50 °C, the maximum activity was found at 30 °C.

### Kinetic analyses of MrSGT

For the kinetic analyses, the production rates of sterol glucosides were plotted vs. the respective substrate concentrations using the SigmaPlot enzyme kinetic module (Systat Software Inc., ver. 12.0, San Jose, CA, USA). All kinetic data are represented as mean ±SD (*n* = 3) relative to the Michaelis–Menten equation. MrSGT reaction kinetics were studied in vitro, with the reaction conditions set at pH 7.5 and 30 °C, and holding the concentrations of the three different sterols (β-sitosterol, campesterol and cholesterol) constant while varying the concentration of UDP-Glc or vice versa. Kinetic parameters such as apparent *K*_m_, *k*_cat_, and *k*_cat_/*K*_m_ values are shown in Table 1. *K*_m_ values for UDP-Glc were all rigid within the range of 0.28–0.33 mM, irrespective of the type of sterol substrate. On the other hand, the enzyme displayed the lowest *K*_m_ value (0.09 mM) for β-sitosterol as the glycosyl acceptor compared to those for campesterol and cholesterol (0.28 and 0.45 mM respectively), demonstrating that MrSGT prefers β-sitosterol as an acceptor to other sterols. Based on the kinetic data obtained (Table 1) and the structural features of the three sterols (Fig. 1), we were able to speculate on the relationship between the substrate structure and the enzyme’s catalytic activity. β-sitosterol and campesterol have the characteristic C-24 side-chains as ethyl or methyl derivatives, respectively, whereas cholesterol does not.

**Table 1:** Kinetic parameters for recombinant MrSGT with UDP-Glc as the glycosyl donor and five different sterols as acceptors

Acceptor	Donor	*k*_cat_ (min^−1^)	*K*_m_ (D) (mM)	*K*_m_ (A) (mM)	*k*_cat_/*K*_m_ (A) (min^−1^ mM^−1^)
β-sitosterol	UDP-Glc	7.31 ± 1.03	0.28 ± 0.03	0.09 ± 0.01	81.2
Campesterol	UDP-Glc	7.66 ± 0.99	0.31 ± 0.04	0.28 ± 0.03	27.4
Cholesterol	UDP-Glc	7.90 ± 1.11	0.33 ± 0.06	0.45 ± 0.05	17.5
Fucosterol	UDP-Glc	7.33 ± 1.64	0.27 ± 0.04	0.13 ± 0.02	56.4
Gramisterol	UDP-Glc	7.83 ± 1.02	0.32 ± 0.05	0.51 ± 0.06	15.4

### Biosynthesis of new PSG derivatives using MrSGT

**Fig. 2:**
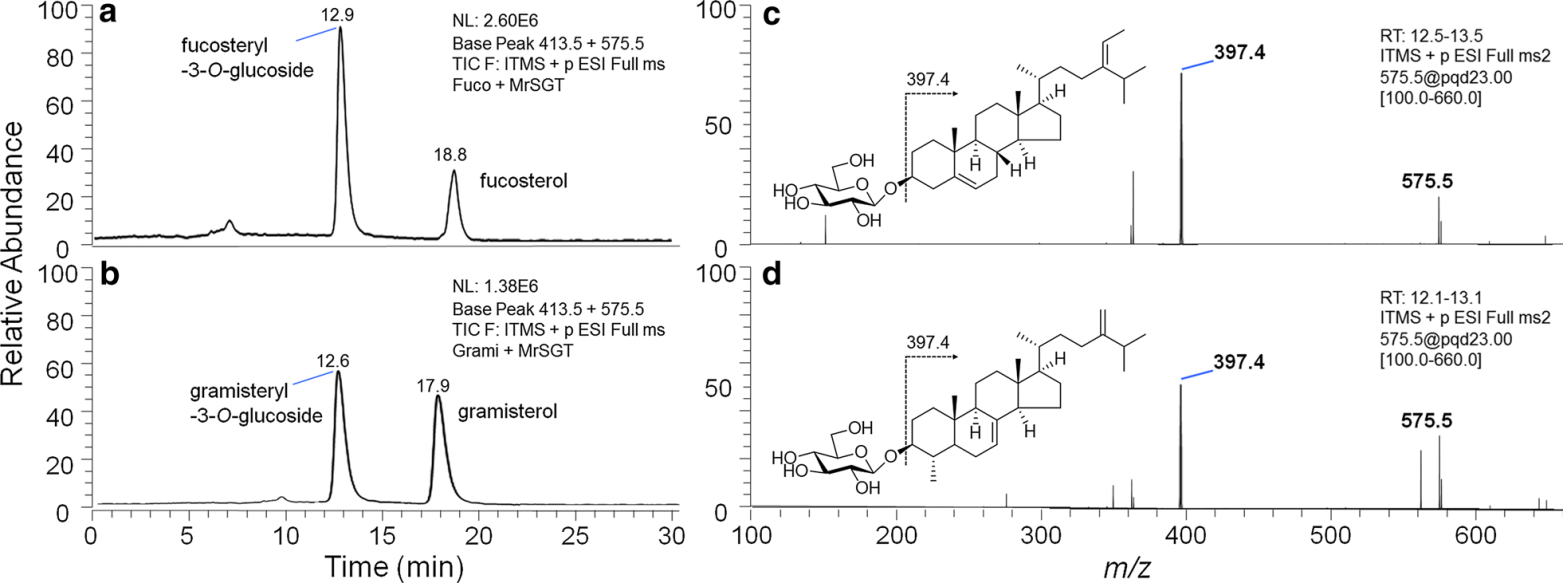
HPLC-MS/MS traces of in vitro MrSGT reactions with **a** fucosterol, **b** gramisterol, and the MS/MS spectra of **c** fucosteryl-3-*O*-glucoside shown as product in chromatogram **a**, **d** gramisteryl-3-*O*-glucoside in chromatogram (**b**)

Additional in vitro MrSGT reactions with other PSs including fucosterol and gramisterol (Fig. 1) were performed to investigate whether the enzyme can catalyze the glucosylation of these two PSs. Fucosterol was chosen as a candidate acceptor in the MrSGT reaction for examining the effect of an altered side-chain (namely, an extra double bond between C-24 and C-28) on the kinetic properties of MrSGT, whereas gramisterol was chosen for studying the effect of further structural differences including a methyl group at C-4 and C-24, 28 methylene group on MrSGT’s catalytic properties. The bioconversion of the above two PSs to their corresponding PSGs was detected using HPLC–MS/MS analyses as in the case of the previously described sterols (Fig. 2) and the kinetic parameters of MrSGT with respect to fucosterol and gramisterol were also determined (Table 1). The *K*_m_ values for fucosterol and gramisterol as glycosyl acceptors averaged at 0.13 and 0.51 mM, respectively, thus demonstrating recombinant MrSGT’s preference for fucosterol as the acceptor over gramisterol. The catalytic efficiency of the enzyme was also significantly different with the two PSs; *k*_cat_/*K*_m_ values were 56.4 and 15.4 min^−1^ mM^−1^ for fucosterol and gramisterol, respectively (Table 1).

### Structural elucidation of fucosterol and gramisterol analogs

After multiple scale-up reactions (>5 batches with fucosterol) followed by MPLC chromatographic isolation, fucosteryl 3-*O*-glucoside could be obtained as a white powder (4.2 mg); HR-ESI–MS ([M + H]^+^*m/z* 575.4233, calcd. 575.4267) and ^1^H- and ^13^C-NMR (see Table 2). HR-ESI–MS of gramisteryl 3-*O*-glucoside (pale powder, 2.7 mg) gave an *m/z* of 575.4242 consistent with the derived formula of C_35_H_58_O_6_ (575.4267). Its chemical structure was further elucidated by NMR spectroscopic analyses (see Table 2).

**Table 2:** ^1^H- and ^13^C-NMR data (500 MHz, DMSO-*d6*) for fucosterol-3-*O*-β-d-glucoside and gramisterol-3-*O*-β-d-glucoside, which were produced by in vitro reaction of recombinant MrSGT together with two glycosyl acceptors (fucosterol and gramisterol) and a glycosyl donor (UDP-glucose), respectively

Position	Fucosterol-3-*O*-β-d-glucoside		Gramisterol-3-*O*-β-d-glucoside	
	δ_H_ (J in Hz)	δ_C_	δ_H_ (J in Hz)	δ_C_
1	1.38 m; 1.13 m	37.3	1.57 m; 1.31 m	37.3
2	1.54 m; 1.29 m	29.7	1.70 m; 1.43 m	28.0
3	2.86 d	81.9	2.77 d	90.0
4	2.23 m; 1.96 m	39.9	1.93 m	39.1
5	–	140.8	1.45 m	52.2
6	5.34 d	121.8	2.04 m; 1.78 m	31.5
7	2.04 m; 1.77 m	32.2	5.36 d	117.4
8	1.45 m	31.8	–	139.1
9	1.42 m	50.7	1.43 m	49.9
10	–	37.7	–	42.5
11	1.51 m; 1.27 m	21.1	1.42 m; 1.19 m	21.1
12	1.58 m; 1.31 m	39.6	1.33 m; 1.09 m	39.4
13	–	42.7	–	44.9
14	1.40 m	56.7	2.15 m	55.1
15	1.62 m; 1.36 m	26.3	1.62 m; 1.38 m	23.6
16	1.60 m; 1.32 m	25.7	1.60 m; 1.35 m	27.4
17	1.47 m	56.1	1.49 m	56.4
18	1.06 s	12.1	1.04 s	14.1
19	1.30 s	19.3	1.06 s	14.5
20	1.64 m	36.2	1.64 m	36.1
21	0.97 m	19.6	0.96 m	19.6
22	1.56 m	33.9	1.54 m	33.8
23	1.44 m	27.3	1.96 m	31.1
24	–	146.5	–	156.5
25	2.52 m	34.7	2.51 m	33.5
26	1.09 d	21.6	1.09 d	21.5
27	1.09 d	21.6	1.09 d	21.5
28	5.19 m	115.2	4.90 m	104.8
29	2.01 overlap	13.2	0.93 d	13.3
1′	4.44 d (7.2)	109.9	4.43 d (7.4)	110.0
2′	3.77 m	74.3	3.75 m	74.2
3′	3.49 m	76.9	3.48 m	76.8
4′	3.46 overlap	71.7	3.44 overlap	71.6
5′	3.75 overlap	81.4	3.73 overlap	81.5
6′	3.79 m; 3.53 dt	62.4	3.79 m; 3.51 dt	62.3

## Discussion

The optimal temperature of MrSGT is lower than that of the previously characterized bacterial SGTs (Lebrun et al. 2006; Thuan et al. 2013); and also lower than the 37 °C optimum of the SGTs present in the human pathogen *H. pylori* and marine actinomycete, *S. tropica*. When the reaction was carried out using β-sitosterol as the acceptor and a wide variety of nucleotide sugars (adenosine diphosphate Glc, cytidine diphosphate [CDP]-Glc, CDP-galactose [Gal], guanosine diphosphate [GDP]-Glc, GDP-Gal, thymidine diphosphate [TDP]-Glc, UDP-Gal, UDP-Glc and UDP-glucuronic acid), the glycosylation occurred only in the reaction using UDP-Glc as the glycosyl donor. SGT from *S. tropica* (Thuan et al. 2013) was able to utilize TDP-Glc as well as UDP-Glc as glycosyl donors for the biosynthesis of β-sitosteryl glucoside. Thus, MrSGT differs from the *S. tropica*-derived SGT in its strict substrate specificity with respect to the glycosyl moiety donor.

The thorough kinetic analyses revealed that the transfer of glucose moiety onto a mammalian sterol or cholesterol can be catalyzed by MrSGT, but at a catalytic efficiency (17.5 min^−1^ mM^−1^) of only 20 % of that for β-sitosterol (81.2 min^−1^ mM^−1^). It is, thus, obvious that while MrSGT can utilize the two PSs as well as cholesterol as substrates, β-sitosterol is its preferred substrate. These results are consistent with our previous findings (Hoang et al. 2016) of the highest bioconversion rates obtained from the MrSGT reactions with β-sitosterol, but differ from the findings on other bacterial SGTs (Smith 1971; Lebrun et al. 2006; Thuan et al. 2013) which have been previously characterized. The two SGTs found in the infectious pathogens, *H. pylori* and *Mycoplasma gallinarum,* show absolute substrate specificity to cholesterol, and are not known to catalyze the biosynthesis of PSG. SGT derived from a marine actinomycete, *S. tropica,* was found to strictly function on β-sitosterol.

As indicated by the significant difference in MrSGT’s kinetics with different sterol substrates, the decoration of alkene (or ethylidene) group at the C-24 branch or the degree of hydrophobicity within this region is likely to affect both catalytic efficacy and substrate preference of MrSGT. The three-dimensional structure of MrSGT could provide valuable insights into the protein–ligand (or catalytic site-ligand) interactions.

From the kinetic data (Table 1), the high *K*_m_ and the low *k*_cat_/*K*_m_ values for gramisterol (as 4-methylsterol) indicated that the structural alteration near the glycosylation site (C-3 hydroxyl position) in the sterol backbone had an adverse effect on MrSGT’s catalytic activity. It is evident from these studies that MrSGT can utilize, albeit with different catalytic efficiency, diverse sterols as substrates for the biosynthesis of PSGs, exhibiting its promiscuity towards glycosyl acceptors.

Fucosterol is one of the PSs commonly isolated from edible brown algae, and its chemical structure was elucidated in 1966 (Nes et al. 1966). It exhibits a number of beneficial medicinal properties such as antioxidant, anti-diabetic, anti-cancerous, anti-inflammatory and anti-osteoporotic bioactivities (Abdul et al. 2016). There is only one publication about the glycosylated analog of fucosterol, which reports the presence of fucosteryl glucoside in the neutralized solvent extracts from the tropical lemongrass herb *Cymbopogon citrus* (Olaniyi et al. 1975). However, the structural details of this analog were not elucidated. There are no reports on the enzymatic glycosylation of fucosterol. Gramisterol is known as a 4-methyl PS present mainly in grain germs and kernel and vegetable oils (Jeong et al. 1975). Two recent publications have reported that gramisterol isolated from the rice bran extract displayed not only anti-cancerous activity against the mouse leukemia cell line, but also anti-tumor and immune enhancing activities against acute myelogenous leukemia (Suttiarporn et al. 2015; Somintara et al. 2016). To date, however, there have been also no reports on the nature of its glucoside and the in vivo or in vitro enzymatic biosynthesis of gramisteryl glucoside.

From the listed chemical shifts of NMR spectra acquired from both glucosides (Table 2), the anomeric proton signal (*δ*_H_ 4.43 or 4.44) present in both glucosides demonstrated the axial (β) resonance, same as that found in β-sitosterol-3-*O*-β-d-glucoside, campesterol-3-*O*-β-d-glucoside and cholesterol-3-*O*-β-d-glucoside (Hoang et al. 2016). Moreover, the coupling constant (*J*) within the range of 7.2–7.4 Hz detected at the anomeric proton of both glucosides, represented the 180° dihedral angle between the two coupled protons, assigning apparent β-anomer. Therefore, the chemical structures of the fucosterol and gramisterol analogues were elucidated as fucosterol-3-*O*-β-d-glucoside and gramisterol-3-*O*-β-d-glucoside, respectively. However, these kinds of glucosides with β-conformation are usually the products of plant-origin SGT reactions (Chaturvedi et al. 2011; Saema et al. 2016; Tiwari et al. 2014). The bacterial SGT from *H. pylori* is known to produce glucoside in α-conformation (namely, cholesterol-3-*O*-*α*-glucoside) (Lebrun et al. 2006). There is no detailed stereo-chemical information on the enzymatic products of other bacterial SGTs from *S. tropica* and *M. gallinarum* (Smith 1971; Thuan et al. 2013). Hence, this is the first report on a stereospecific bacterial SGT which can attach a glucose moiety in β-conformation at the C-3 hydroxyl group of sterols.

Using the kinetic analyses of UDP-Glc-dependent MrSGT, we were able to confirm the promiscuity of recombinant MrSGT, in contrast to the previously discovered bacterial SGTs. Exploiting this substrate flexibility, two new sterol glucosides, whose chemical structures have not been described before, were stereo-specifically biosynthesized, and their chemical structures were elucidated as fucosterol-3-*O*-β-d-glucoside and gramisterol-3-*O*-β-d-glucoside, respectively. Glycosylation of PSs with known bioactivities can augment the structural diversity of natural products and make it possible to expand the therapeutic applications of PSs by enhancing the known bioactivities or endowing them with new bioactivities. Further studies on the comparison of the bioactivities of non-glycosylated PSs with those of their PSG derivatives should be the direction of future research in this area.
